# Prevalence and determinants of poor glycaemic control amongst patients with diabetes followed at Vanga Evangelical Hospital, Democratic Republic of the Congo

**DOI:** 10.4102/phcfm.v13i1.2664

**Published:** 2021-04-30

**Authors:** Lino Masingo Cedrick, Jean-Pierre Fina Lubaki, Lepira Bompeka Francois, Ogunbanjo Adebola Gboyega, Lukanu Ngwala Philippe

**Affiliations:** 1 Department of Family Medicine, Faculty of Medicine, Protestant University of Congo, Kinshasa, Democratic Republic of the Congo; 2 Department of Family Medicine and Primary Health Care, Faculty of Medicine, Protestant University of Congo, Kinshasa, Democratic Republic of the Congo; 3 Department of Nephrology, Faculty of Medicine, University of Kinshasa, Kinshasa, Democratic Republic of the Congo; 4 Department of Family Medicine and Primary Health Care, Faculty of Medicine, Sefako Makgatho Health Sciences University, Pretoria, South Africa

**Keywords:** determinants, diabetics, glycaemic control, rural, poor, prevalence, patients

## Abstract

**Background:**

The prevalence of diabetes mellitus is increasing dramatically in developing countries, where diabetic patients usually present with poor glycaemic control, leading to complications and worsening the prognosis.

**Aim:**

The aim of this study was to determine the extent of poor glycaemic control and its determinants in diabetic patients.

**Setting:**

The study was conducted in a rural area of the province of Kwilu, Democratic Republic of the Congo.

**Methods:**

This research comprised a cross-sectional study involving 300 Type 1 and 2 diabetic patients attending Vanga Evangelical Hospital in the Democratic Republic of the Congo from January 2018 to March 2018. Patients’ sociodemographic, clinical and biological characteristics, accessibility to the health structure and treatment were described. The determinants of poor glycaemic control were identified using multivariate logistic regression at the *p* < 0.05 level of statistical significance.

**Results:**

The mean age of participants was 46.9 ± 16.3 years, 68.4% were men, and 62.3% had Type 2 diabetes mellitus. Poor glycaemic control was present in 78% of patients. The independent determinants of poor glycaemic control were tobacco use (adjusted odds ratio [aOR]: 2.01 [1.77–5.20], *p* = 0.015), the presence of comorbidities (aOR: 2.86 [1.95–6.65], *p* = 0.007), the presence of a factor contributing to hyperglycaemia (aOR: 2.74 [1.83–3.67], *p* = 0.014), missing scheduled appointments (aOR: 2.59 [1.94–7.13], *p* = 0.006) and non-adherence to treatment (aOR: 4.09 [1.35–6.39], *p* = 0.008).

**Conclusion:**

This study shows that more than three-quarters of diabetics undergoing treatment are not controlled, with mainly patient-related factors as the main explanatory factors for this poor glycaemic control. Therefore, the establishment of a therapeutic education programme and wider integration of diabetes care services, mainly at the primary level of the healthcare pyramid, should contribute to improved diabetes treatment.

## Introduction

In 2014, the World Health Organization estimated that 422 million people were living with diabetes, a prevalence that had almost doubled from 4.7% in 1980 to 8.5% in 2014.^1^ According to the International Diabetes Federation (IDF), the number of people living with diabetes will increase in Africa from 14.2 million in 2015 to 34.2 million in 2040^2^; demographic and nutritional transitions, as well as rapid and unplanned urbanisation, are some of the explanatory factors for this development.^3,4^ In sub-Saharan Africa, the number of diabetic patients was estimated at 14.7 million in 2011, with 737 090 cases in the Democratic Republic of the Congo (DRC).^5^ In Kinshasa, a study conducted in 2000 estimated the prevalence of diabetes to 7% in adults.^6^ In Kisantu, a semi-urban area in the DRC, the prevalence of diabetes mellitus was estimated at 4.8% in 2007.^7^ In Bukavu, Katchunga et al. reported a prevalence rate of 7.3% in 2012.^8^

Adequate glycaemic control is recommended in both type 1 and type 2 diabetic patients to delay the onset of complications,^9,10^ improve the quality of life of patients and at the same time reduce the high cost of diabetes care for the health sector.^7,8,10^ However, as it is with other chronic diseases, very few diabetic patients consult on time to obtain optimal medical intervention and treatment for their diseases because of multiple factors.^11^ The prevalence of poor glycaemic control varies, from 29% to 73% in diabetic patients aged greater than or equal to 65 in the United States (US),^12^ 65% in France,^13^ 71% in East, West and Central Africa^14^ and 68% in the DRC.^15^ Many factors determine poor glycaemic control in patients, related to diabetes disease and sociodemographic, clinical and treatment characteristics; access to quality care and patient adherence to follow-up recommendations and lifestyle change also play a significant role.^16,17^ Mbanya et al. found, in the International Diabetes Management Practices Study (IDMPS) in 12 African countries, that unhealthy diet, lack of exercise, lack of self-management and hospitalisation in the past 12 months were predictors of poor glycaemic control in both types of diabetes, whilst poor diabetes education and longer duration of diabetes were predictors of poor glycaemic control for only Type 1.^18^

In 2009, a study conducted in Vanga amongst diabetic patients showed that only 34.4% had optimal or acceptable glycaemic control.^19^ Complications of diabetes are a common reason for outpatient consultations or provision of emergency services at Vanga Evangelical Hospital. For example, a study on ocular complications of diabetes conducted in Vanga in 2014 found that 177 participants (63.2%) had at least one ocular complication.^20^ Factors associated with poor glycaemic control vary throughout settings. The objective of this study was to assess the extent of poor glycaemic control and its determinants in type 1 and 2 diabetic patients attending Vanga Evangelical Hospital.

## Methods

### Type of study

This was a cross-sectional study.

### Study period and setting

The study took place from 01 January 2018 to 31 March 2018 at the Vanga Evangelical Hospital–the Referral Hospital of the Rural Health District of Vanga in the province of Kwilu, in the south-west of the DRC. The health district of Vanga includes 55 health facilities spread over an area of 2600 km^2^. The Vanga Evangelical Hospital is the sole facility in the area that runs a diabetic clinic. The clinic runs twice a week, providing outpatient services to diabetic patients including education, examination and treatment. The clinic has 1025 registered patients. The average attendance per clinic day was 50 patients. A team of two doctors, three nurses, a pastor and a social worker operate the diabetic clinic.

### Study population

The study population consisted of diabetes mellitus patients attending the Diabetic Clinic of Vanga Evangelical Hospital.

#### Selection criteria

All patients with diabetes Type 1 and Type 2, aged 15 years or more, attending the outpatient diabetic clinic for 6 months or longer were eligible for the study.

#### Sample size

The sample size was estimated using the Fischer formula of a known population of patients with diabetes (1025), taking into account a 95% confidence interval (CI) with an expected accuracy of 5% and a poor glycaemic control rate of 65.6%. The minimum size of 230 patients was estimated. A total of 300 participants were selected.

#### Sampling

Selection of the participants was done through systematic sampling. The sampling frame was calculated as *k* = 1025/300 or 3. The starting point for inclusion was a number chosen between 1 and *k*; the number 1 was chosen. At each session, a list of patients was drawn up in order of arrival at the clinic. On this list, on each clinic day, the first patient of the day was included in the study, and every third patient after that was included. If the first and third patients were ineligible, the next patient on the list was selected. At each session, the principal researcher or the research assistants interviewed a maximum of four patients. The process was repeated until the desired sample size was attained.

### Data collection

The principal investigator and three trained research assistants performed the data collection. Prior to patients’ inclusion in the study, members of the research team explained the purpose and objectives of the study to the mand obtained informed consent. All patients were interviewed with a structured and pretested questionnaire and given a physical examination (performed by the principal investigator with the help of the Vanga Hospital medical team). Early morning blood capillary samples were taken to measure glycosylated haemoglobin (HbA1c) using spectrophotometer from Shenzhen Genius Electronics (Shenzhen, China). The variables of interest in the study were sociodemographic characteristics, accessibility to the health structure, diabetes characteristics and treatment modalities. Poor glycaemic control was defined by a level of glycosylated HbA1c of ≤ 7.0%, whilst adequate glycaemic control was defined by an HbA1C level of > 70% and complications and comorbidities considered in the study were those described for patients’ record booklets.

### Statistical analysis

The data were encoded using Excel 2010 software; after checking and cleaning the database, the data were exported to Statistical Package for Social Sciences (SPSS) software version 21 (Chicago, IL, US). The statistical data analysis consisted of calculating the means or medians and interquartile ranges (IQRs) or standard deviation (SD) for continuous variables and the frequencies (*n*) and percentages (%) for categorical variables. For bivariate analysis, continuous variables were transformed to categorical variables (age, duration of the disease, monthly income, household size). Pearson’s chi-square or Fischer’s exact test were used to compare proportions. Logistic regression was used to assess the determinants of poor glycaemic control; odds ratios (ORs) and their 95% CIs were used to estimate the strength of association between the independent and dependent variables. A value of *p* ˂ 0.05 was set as the statistical significance threshold.

### Ethical considerations

Ethics approval was granted by the ethics committee of the Protestant University of Congo (CEUPC0035).

The protocol was submitted to and approved by the Ethics Committee of the Protestant University in Congo (approval no. CUPC0047). Authorisation to conduct the study was obtained from the hospital authorities. The participants were asked to sign an informed consent form after they were given an explanation of the purpose and objectives of the study and were offered an opportunity to ask any questions regarding the conduct of the study. The participants were assured of their right to withdraw from the study at any time they wished without having to give a reason and without any negative consequences for the care they received at the diabetic clinic. The anonymity of patients and confidentiality of their data were guaranteed at all times.

## Results

The study sample consisted of 300 patients with diabetes, out of 314 patients asked to participate in the study (yielding a non-response rate of 4.5%). Men represented 68.4% of participants, and Type 2 diabetes was more prevalent. The mean age of participants was 46.9 ± 16.3 years. The majority of participants were in the 40–59-year age group (44.7%), married (72%), had not attained university education (91.7%) and had low income (56.0%). The mean distance to reach the health centrewas 32 km by foot forthe majority of participants (74.7%). The mean duration of the disease for the majority of respondents was less than 10 years (78.7%). Alcohol intake and tobacco use were noted for 22.7% and 13.7% of participants, respectively. More than a quarter of the participants (27.3%) had abdominal obesity, and 19.3% had hypertension. Nearly one-third of diabetic patients (34.0%) had a factor contributing to hyperglycaemia, amongst which infection was the most prevalent (20%). Insulin (65.0%) was the most common treatment, followed by glibenclamide (29.0%) and metformin (26.0%). Fourteen percent of the patients attended the clinic two or more times every month, and 92% felt they respected the appointment schedules. Seventy-nine percent of participants felt that they had strictly followed the instructions for taking medication. Poor glycaemic control was observed in 78% of patients. When comparing the two patient groups constituted by those with adequate versus those with poor glycaemic control in terms of sociodemographic characteristics, only income less than 60 United States dollars (USD) was significant for poor glycaemic control (42.4% vs. 63.2%, *p* = 0.019) (Table 1).

**TABLE 1:** Participants’ sociodemographic characteristcics and poor glycaemic control, Vanga, 2018.

Variable	Poor glycaemic control		Adequate glycaemic control		*p*
	*n*	%	*n*	%	
**Age (years)**					0.460
15–39	73	31.2	25	37.9	-
40–59	109	46.6	25	37.9	-
60–88	52	22.2	16	24.2	-
**Sex**					0.209
Man	160	68.4	41	62.1	-
Woman	74	31.6	25	37.9	-
**Occupation**					0.594
Unemployed	44	18.8	16	24.2	-
Independent	131	56.0	36	54.5	-
Official	59	25.2	14	21.2	-
**Monthly income**					0.019
< 60 SD	148	63.2	28	42.4	-
≥ 60 SD	86	36.8	38	57.6	-
**Educational level**					0.636
No level	23	9.8	3	4.5	-
Primary	65	27.8	19	28.8	-
Secondary	126	53.8	38	57.6	-
University	20	8.5	6	9.1	-
**Household size**					0.319
≤ 6	168	71.8	50	75.8	-
> 6	66	28.2	16	24.2	-

Note: The data are expressed as the mean ± standard deviation, frequency (absolute) and relative (in percentage).

*N* = 300.

SD, standard deviation.

Accessibility to the health centre and all its components was not associated with poor glycaemic control (Table 2).

**TABLE 2:** Accessibility to the health centre and poor glycaemic control amongst diabetic patients, Vanga, 2018.

Variable	Poor glycaemic control		Adequate glycaemic control		*p*
	*n*	%	*n*	%	
**Means of transport**					0.121
Motorcycle	38	16.2	6	9.1	-
Foot	175	74.8	49	74.2	-
Vehicle	4	1.7	3	4.5	-
Bicycle	14	6.0	8	12.1	-
Canoe	3	1.3	0	0.0	-
**Time taken to reach health facility (hours)**					0.724
0–1	57	24.4	12	18.2	-
2–5	76	32.5	24	36.4	-
6–24	80	34.2	25	37.9	-
> 24	21	9.0	5	7.6	-
**Frequency of visits**					0.594
Frequent	119	50.9	33	50.0	-
Occasional	101	43.2	31	47.0	-
Non-existent	14	6.0	2	3.0	-

Note: The data are expressed as frequency (absolute) and relative (in percentage).

*N* = 300.

When considering the clinical parameters and treatment characteristics (Table 3), patients with poor glycaemic control comprised a significantly higher proportion of those who were consuming alcohol (26.5% vs. 9.1%, *p* = 0.022), smokers (15.0% vs. 9.1%, *p* = 0.015), had a diabetes duration of ≥ 10 years (26.1% vs. 4.5%, *p* < 0.001), had Type 2 diabetes (65.8% vs. 50.0%, *p* = 0.035) and had comorbidities (31.6% vs. 15.2%, *p* = 0.017), with mainly hypertension (23.5% vs. 4.5%, *p* < 0.001) and heart disease (17.1% vs. 1.5%, *p* = 0.039). They also comprised a significantly higher proportion of poor compliant patients (23.5% vs. 4.5%, *p* < 0.001) and those with fewer than two visits per month (92.7% vs. 62.1%, *p* = 0.008). As patients with Type 1 and Type 2 diabetes showed a difference in diabetes control, we conducted a comparison of these two kinds of patients that showed the difference by level of education (*p* = 0.03) and profession (*p* = 0.028).

**TABLE 3:** Clinical characteristics, treatment modalities and poor glycaemic control amongst diabetic patients, Vanga, 2018.

Variable	Poor glycaemic control		Adequate glycaemic control		*p*
	*n*	%	*n*	%	
Alcohol intake	62	26.5	6	9.1	0.022
Tobacco consumption	35	15.0	6	9.1	0.015
**Duration of diabetes (years)**					< 0.001
< 10	173	73.9	63	95.5	-
≥ 10	61	26.1	3	4.5	-
**Type of diabetes**					0.035
Type 1	80	34.2	33	50.0	-
Type 2	154	65.8	33	50.0	-
**Comorbidities**	74	31.6	10	15.2	0.017
Hypertension	55	23.5	3	4.5	< 0.001
Heart disease	4	17.1	1	1.5	0.039
Diabetic retinopathy	3	1.3	2	3.0	0.304
Tuberculosis	7	3.0	3	4.5	0.383
Chronic kidney disease	3	1.3	1	1.5	0.632
Overweight	43	18.4	13	19.7	0.466
Obesity	11	4.7	4	6.1	0.428
**Factors contributing to hyperglycaemia**					
Infection	50	21.4	10	15.2	0.042
Poor adherence	221	94.4	50	75.8	< 0.001
Diabetic retinopathy	3	1.3	0	0.0	-
**Treatment received**					
Insulin	157	67.1	39	59.1	0.145
Glibenclamide	63	26.9	24	36.4	0.092
Metformin	55	23.5	23	34.8	0.047
Regular intake of treatment	226	96.6	65	98.5	0.375
Session deemed useful	225	96.2	66	100.0	0.103
**Frequency of visits/month**					0.008
< 2	217	92.7	41	62.1	-
≥ 2	17	7.3	25	37.9	-

*N* = 300.

Multivariate analysis by logistic regression retained a model with the following independent factors of poor glycaemic control: smoking (adjusted OR [aOR]: 2.01 [1.77–5.20], *p* = 0.015), the presence of comorbidities (aOR: 2.86 [1.95–6.65], *p* = 0.007), the presence of a factor contributing to hyperglycaemia (aOR: 2.74 [1.83–3.67], *p* = 0.014), the non-respect of appointment schedules (aOR: 2.59 [1.94–7.13], *p* = 0.006) and non-compliance to treatment (aOR: 4.09 [1.35–6.39], *p* = 0.008) (Table 4).

**TABLE 4:** Determinants of poor glycaemic control in the multivariate analysis.

Variable	Univariate analysis			Multivariate analysis		
	*p*	OR	95% CI	*p*	aOR	95%CI
**Monthly income (USD)**						
≥ 60	-	-	-	-	-	-
< 60	0.031	3.73	1.60–8.75	0.963	1.01	0.56–1.82
**Alcohol intake**						
No	-	-	-	-	-	-
Yes	0.029	3.12	1.59–5.13	0.277	1.46	0.73–2.92
**Tobacco use**						
No	-	-	-	-	-	-
Yes	0.023	2.76	1.71–4.38	0.015	2.01	1.77–5.20
**Duration of diabetes (years)**						
< 10	-	-	-	-	-	-
≥ 10	0.013	3.14	1.58–6.25	0.668	1.17	0.57–2.43
**Type of diabetes**						
Type 1	-	-	-	-	-	-
Type 2	0.028	3.17	1.66–5.07	0.982	1.01	0.55–1.86
**Presence of comorbidities**						
No	-	-	-	-	-	-
Yes	0.028	2.39	1.77–3.50	0.007	2.858	1.95–6.65
**Factors contributing to hyperglycaemia**						
No	-	-	-	-	-	-
Yes	0.027	2.52	1.73–3.20	0.014	2.74	1.83–3.67
**Respect for appointments**						
Yes	-	-	-	-	-	-
No	0.010	4.29	1.86–6.09	0.006	2.59	1.94–7.13
**Compliance to treatment**						
Yes	-	-	-	-	-	-
No	0.008	4.09	1.42–2.80	0.008	4.09	1.35–6.39

aOR, adjusted odds ratio; CI, confidence interval; OR, odds ratio; USD, United States dollars.

## Discussion

More than three-quarters of our participants had poor glycaemic control. The independent determinants of poor glycaemic control were smoking, the presence of comorbidities, the presence of a factor contributing to hyperglycaemia, non-respect of appointment schedules and non-adherence to treatment.

The prevalence of poor glycaemic treatment, based on the glycosylated haemoglobin assay, was 78%. Our observation corroborates that made by several authors, notably in Brazil and Africa.^20,21^ It is lower than the 86% reported by Gebre-Yohannes and colleagues in Ethiopia.^22^ On the other hand, it is higher than the 60.5% and 68.0% found by Longo-Mbenzaand colleagues and Otienoand colleagues in the DRC and Kenya, respectively.^15,23^ The disparity in the prevalence between studies may be explained by the differences in sample size, the methodology and the populations’ characteristics, as well as the definitions of glycaemic treatment and the statistical methods used.

In our study, smoking was a determinant of poor glycaemic control. Diabetics with a history of smoking were twice more likely to have poor glycaemic control than non-smokers. Our observation is similar to that of Melinand colleagues^24^; however, the association of smoking with poor glycaemic control is not consistent in all available studies.^25,26^ Tobacco may negatively affect glycaemic control by stimulating the sympathetic system responsible for glycogenolysis and gluconeogenesis^27^ and stimulating the renin–angiotensin system on the basis of post-receptor insulin resistance.^28^

The presence of comorbidities was a determinant of poor glycaemic control in our study; patients with comorbidities were almost three times as likely to be uncontrolled compared to those without comorbidities. It is established that the presence of comorbidities causes stimulation of the production of counter-regulation hormones, particularly cortisol, through the stress they induce.^29^

Non-respect of scheduled appointments was associated with poor glycaemic control, increasing the risk of poor control by three in our sample. Rhee and colleagues showed that diabetic patients with at least one annual visit were more adherent to their medication and had an average HbA1c lower (7.6%) compared to those without annual visits (HbA1c: 9.7%).^30^

In Venezuela, satisfaction with the treatment of diabetes was associated with good glycaemic control in a study done by Moreira and colleagues^31^ but not with attendance in a therapeutic education programme. Medical monitoring strengthens and contributes to the self-management maintenance of diabetics, thus promoting better glycaemic control.

In our study, patients not adhering to treatment were four times more likely to have poor glycaemic control compared to those adhering. Our observation corroborates that of Kakoma and colleagues,^32^ who, in a study conducted in Lubumbashi, DRC, reported poor adherence as the main factor in the decompensation of diabetes (29.4%).^30^

The presence of a factor contributing to hyperglycaemia is a determinant of poor glycaemic control, which multiplies this risk by three. This observation corroborates that made by Maâlej and colleagues of a strong association between the imbalance of diabetes and the presence of a factor contributing to hyperglycaemia, in particular, pulmonary infection with a multiplication of the risk of occurrence of two to six times.^33^ The infection represents stress to the body, and the stress response leads to the activation of the sympathetic system with the release of hyperglycaemic catecholamines.^28^

Diabetes is increasing in importance in our settings with few resources for health and health systems directed to other priorities; working to achieve an adequate glycaemic control for diabetic patients through cost-effective interventions is crucial. Most of the factors identified in our study, such as smoking, adherence to treatment and respect of schedules, could be modified through effective education.

The interpretation of our results must take into account some limitations. Not all factors recognised as being important predictors of glycaemic control in Africa have been assessed in this study, for example diet, exercise and diabetes education.^18^ As a cross-sectional study, it cannot establish a cause-and-effect relationship between the different variables of interest. The small size of the sample does not allow identification of the additional associations between the different variables of interest. The unique HBA1c assay should, given the variability of biological parameters, under- or overestimate the prevalence of poor glycaemic control. The hospital setting does not make it possible to generalise the results of this study to all patients with diabetes in Vanga. Nevertheless, the strength of this study is having generated preliminary data on glycaemic control in a rural environment, which often is not taken into account in health programmes because of the lack of data.

Future research should look for other factors related to glycaemic control, such as genes or therapeutic schemes available, and moreover define health strategies to improve control amongst diabetic patients.

## Conclusion

This study showed that more than three-quarters of diabetics undergoing treatment are not controlled, with patient-related factors as the main explanatory factors for this poor glycaemic control. Therefore, the establishment of a therapeutic education programme and wider integration of diabetes care services mainly at the primary level of the healthcare pyramid should contribute to improved diabetes treatment.
